# Epidemiologic trends in cancer-related emergency department utilization in Korea from 2015 to 2019

**DOI:** 10.1038/s41598-021-01571-1

**Published:** 2021-11-09

**Authors:** Sun Young Lee, Young Sun Ro, Sang Do Shin, Sungwoo Moon

**Affiliations:** 1 Public Healthcare Center, Seoul National University Hospital, Seoul, Korea; 2 Laboratory of Emergency Medical Services, Seoul National University Hospital Biomedical Research Institute, Seoul, Korea; 3 Department of Emergency Medicine, Seoul National University Hospital, Seoul, Korea; 4 National Emergency Medical Center, National Medical Center, Seoul, Korea; 5 Department of Emergency Medicine, Korea University Ansan Hospital, Gyeonggi, Korea

**Keywords:** Health care, Oncology

## Abstract

It is inevitable for cancer patients to visit the emergency department (ED) for symptoms of cancer itself and various treatment-related complications. As the prevalence of cancer increases along with cancer survival rates, the number of ED visits of cancer patients may increase. This study aimed to investigate the epidemiologic trends and characteristics of cancer-related ED visits. A cross-sectional study was conducted for all ED visits nationwide between 2015 and 2019. The characteristics of cancer- and non-cancer-related ED visits were compared, and the cancer type and primary reason for ED visits were investigated for cancer-related ED visits. The age- and sex-standardized incidence rate per 100,000 population was calculated. Among 44,983,523 ED visits for 5 years, 1,372,119 (3.1%) were cancer-related. Among cancer-related ED visits, 54.8% led to hospitalization including 5.1% in ICU, and 9.5% died in the hospital. The age- and sex-standardized incidence rates of cancer-related ED visits per 100,000 population increased from 521.8 in 2015 to 642.2 in 2019 (p-for-trends, < 0.01), and rates of cancer-related hospital admission via ED were 309.0 in 2015 and 336.6 in 2019 (p-for-trends, 0.75). The most common cancer types were lung cancer (14.7%), liver cancer (13.1%), and colorectal cancer (11.5%). The most common primary reasons of cancer-related ED visits were pneumonia (3.6%), gastroenteritis (2.7%), fever (2.6%), abdominal pain (2.4%), and ileus (2.1%). Cancer-related ED visits accounted for 3.1% of all ED visits, with 1.37 million cases over five years. The incidence rate of cancer-related ED visits has increased year by year, with high hospitalization and mortality rates, and the burden of cancer-related ED visits will continue to increase as the prevalence increases.

## Introduction

Cancer is an important public health burden globally^1^. Around the world, the 5-year prevalence of cancer reaches 50 million people, and 19 million new cases occur annually^2^. Cancer patients frequently visit hospitals for complex cancer treatments such as chemotherapy and radiation therapy, as well as various problems that occur during care^3^. In case of sudden worsening of symptoms or side effects of cancer treatment after the end of inpatient treatment, it is inevitable for cancer patients to visit the emergency departments (EDs)^4^. The ED is an important medical resource for patients with acute medical illnesses that requires a lot of manpower and equipment to operate^5^.

The number of patients visiting the ED is increasing rapidly, especially in countries with an aging population and high prevalence of chronic diseases^6,7^. EDs should be prepared to provide adequate care in response to emergency diseases where timely treatment is critical for prognosis, such as acute myocardial infarction and severe injury^5,8^. At the same time, non-urgent treatment for chronic diseases or ambulatory care-sensitive conditions is also provided in the ED, due to the nature of the ED operating 24 h a day, 7 days a week^5,9^. As such, it is necessary to monitor the characteristics of patients visiting the ED in order to ensure that the ED can function properly for patients with emergency conditions.

Cancer patients are bound to visit the ED for symptoms caused by the disease itself and side effects from cancer treatment^4,10^. Cancer-related ED visits accounted for 3.7 to 4.2% of all ED visits^10,11^. Approximately 27% of patients with solid tumors who received chemotherapy had at least one visit to ED^4^. Cancer patients stayed in the ED longer, received more radiological tests, were more likely to present acute care conditions in the ED, and were hospitalized more after treatment, compared to non-cancer patients^10–12^. Cancer is not a traditional emergency disease to be treated in the ED as it is a chronic disease^13^. Nevertheless, cancer patients have no choice but to visit the ED sometimes, and use a lot of ED resources when they do. However, not much is known about characteristics of cancer-related ED visits.

As the prevalence of cancer increases along with cancer survival rates, the number of ED visits of cancer patients may increase. However, it is not known what proportion of cancer patients visit the ED nor why. Research on cancer-related ED visits is important both for purposes to improve the quality of cancer treatment by understanding the clinical needs of cancer patient, and for reducing unnecessary ED visits by investigating the epidemiologic characteristics of cancer-related ED visits^10^. The aim of this study is to investigate the epidemiologic trends and characteristics of cancer-related ED visits using a nationwide ED database. In detail, we aimed to investigate the scale and disposition of ED visits according to the cancer type and primary cause of ED visits, and explore factors associated with intensive care unit (ICU) admission of cancer-related ED visits.

## Methods

### Study design and data sources

A cross-sectional study was conducted with the National Emergency Department Information System (NEDIS) database. NEDIS is a nationwide ED based database constructed by the Ministry of Health and Welfare in 2013 and operated by the National Emergency Medical Center. NEDIS collected clinical and administrative information of patients visiting EDs from a total 402 EDs across the country in 2019^14^. All patient-related information including demographics, prehospital, ED, and disposition information was automatically transferred from each ED to a central government server within 14 days of the discharge date from the ED or hospital. The trained coordinators designated in each institution managed the data uploading process.

### Study setting

Korea has a population of approximately 50 million people in 17 provinces including eight metropolitan cities. National Health Insurance (NHI) was implemented in Korea in 1989. NHI is a single insurer operated by Korean government covering the entire population. Since NHI covered all inpatient, outpatient, and emergency care, Korea has high accessibility to medical care including emergency care^15,16^. In addition, due to NHI’s policy of expanding benefit coverage for serious diseases, cancer patients have a lower rate of out-of-pocket expenditure and have higher access to medical care^17^. Under this healthcare system, EDs are open to all beneficiaries without restriction^15^. EDs are classified into 3 levels according to resource and capacity, which are defined by the Ministry of Health and Welfare. In 2020, 38 regional EDs (Level 1), 125 local EDs (Level 2), and 239 emergency facilities (Level 3) were operated.

The National Cancer Center manages the Korea Central Cancer Registry (KCCR) in accordance with the Cancer Control Act, and reports an “Annual report of cancer incidence, survival and prevalence in Korea” since 2007^18^. As the population ages, the incidence and prevalence of cancer in Korea are on the rise. In 2017, there were 0.23 million newly diagnosed cancer cases, and 5-year cancer prevalence was 1.87 million^18^.

### Study population

The study population was patients who visited EDs from January 2015 to December 2019. Cases with unknown age were excluded. The NEDIS data had multiple diagnosis codes (up to 40) at the time of discharge from the ED or hospital using the International Classification of Diseases, 10th edition (ICD-10). The term cancer-related ED visit was defined as if any of the diagnosis codes entered in NEDIS database were ICD-10 code C00–C97 (malignant neoplasms).

### Outcome measures and variables

The study outcomes were ICU admission, hospital admission via ED, and in-hospital mortality.

We obtained the following information from the NEDIS database: patients’ demographics (age, sex, and insurance types (NHI , Medical Aid, and others)), prehospital and ED information (region of ED (metropolitan or not), route of ED visit (direct, transfer-in, and others), use of EMS when visiting ED, ED visit times, level of ED (level 1, 2 and 3)), ED disposition (ED discharge diagnosis code and disposition status (discharge, transfer-out, ward admission, ICU admission, death, and else), and hospital disposition in case of hospitalization (hospital discharge diagnosis code and disposition status (discharge, transfer-out, death, and else)).

Cancer type was classified based on the ICD-10 code^18^. If multiple cancer codes were recorded in one visit, it was classified as the cancer type of the code listed in the previous order. The primary reason of ED visits of cancer patients was defined as non-cancer diagnosis codes among diagnosis codes of NEDIS database. Cases with only cancer diagnosis were excluded from the analysis of the primary reason of ED visits. If multiple non-cancer diagnoses were entered, the first listed diagnosis code was used for the analysis.

### Statistical analysis

Descriptive analysis was conducted to compare the characteristics of cancer-related and non-cancer-related visits in ED. Categorical variables were shown by counts and proportion and tested by Chi-square test. Continuous variables were shown by medians and quartiles and tested by Wilcoxon rank-sum test.

To investigate the annual trends of cancer-related ED visits, the crude and the age- and sex-standardized incidence rates of cancer-related ED visits and study outcomes per 100,000 population were calculated using a direct standardization method using the 2020 mid-year census population as a standard population^19^. Yearly trends of age- and sex-standardized incidence rates were tested by p-for-trend test for a total of cancer-related ED visits and top 5 cancer-types.

Among cancer-related ED visits, a multivariable logistic regression analysis was conducted to investigate associated factors with ICU admission. Adjusted odds ratios (AORs) and confidence intervals (CIs) were calculated. Data management and statistical analyses were conducted using SAS software version 9.4 (SAS Institute Inc., Cary, NC, USA) and Stata version 13.1 (StataCorp, College Station, TX). Statistical significance was taken as P < 0.05.

### Ethical statement

This study was approved by the Institutional Review Boards of Seoul National University Hospital (approval No. SNUH-2012-104-1183), and the requirement for informed consent was waived due to the retrospective nature of this study. All methods were performed in accordance with relevant guidelines and regulations.

### Patient and public involvement statement

The National Emergency Medical Center under the Ministry of Health and Welfare was involved in the design and conduct of this research, but it was not possible to involve patients in our research.

## Results

### Characteristics of the study population

During the 5 years of study period, the total number of ED visits was 44,984,017. Excluding 494 cases of unknown age, 44,983,523 cases were included in the study.

Among them, there were 1,372,119 cancer-related ED visits, which accounted for 3.1% of all ED visits. The median age of cancer-related visits was 65 years old, older than that of non-cancer-related visits (41 years old). Among cancer-related ED visits, 58.0% visited metropolitan ED and 18.1% used EMS. A total of 54.8% of cancer-related visits were hospitalized, and 5.1% were admitted to the ICU, which were higher than those of non-cancer-related ED visits (hospital admission 16.8% and ICU admission 2.3%). The in-hospital mortality of cancer-related ED visits was 9.5%, which was higher than 1.0% of non-cancer-related ED visits (Table 1).

**Table 1:** Demographics of cancer-related and non-cancer-related emergency department visits from 2015 to 2019.

	Total		Non-cancer		Cancer	
	N	%	N	%	N	%
Total	44,983,523	100.0	43,611,404	100.0	1,372,119	100.0
**Age, year**						
0–18	10,200,978	22.7	10,172,301	23.3	28,677	2.1
19–64	25,672,168	57.1	25,035,938	57.4	636,230	46.4
65–120	9,110,377	20.3	8,403,165	19.3	707,212	51.5
Median (IQR)	42 (21–60)		41 (20–60)		65 (55–75)	
Sex, female	21,790,304	48.4	21,224,004	48.7	566,300	41.3
**Insurance**						
National Health Insurance	39,171,555	87.1	37,938,191	87.0	1,233,364	89.9
Medical Aid	2,611,816	5.8	2,486,013	5.7	125,803	9.2
Others	3,200,152	7.1	3,187,200	7.3	12,952	0.9
Metropolitan	19,781,628	44.0	18,986,485	43.5	795,143	58.0
**Route of ED visit**						
Direct	41,141,931	91.5	40,107,509	92.0	1,034,422	75.4
Transfer-in	3,186,182	7.1	2,955,923	6.8	230,259	16.8
Others	655,410	1.5	547,972	1.3	107,438	7.8
EMS use	6,998,753	15.6	6,750,443	15.5	248,310	18.1
ED visit time						
Night	23,241,110	51.7	22,799,287	52.3	441,823	32.2
Weekend	17,133,056	38.1	16,762,081	38.4	370,975	27.0
**Level of ED**						
Level 1	8,248,885	18.3	7,835,584	18.0	413,301	30.1
Level 2	16,922,243	37.6	16,695,909	38.3	226,334	16.5
Level 3	19,812,395	44.0	19,079,911	43.7	732,484	53.4
**Disposition of ED**						
Discharge	35,616,831	79.2	35,057,229	80.4	559,602	40.8
Transfer-out	768,668	1.7	721,068	1.7	47,600	3.5
Admission, total	8,096,264	18.0	7,343,973	16.8	752,291	54.8
Death	241,129	0.5	230,036	0.5	11,093	0.8
Others	260,631	0.6	259,098	0.6	1,533	0.1
Admission, ICU	1,092,039	2.4	1,022,091	2.3	69,948	5.1
In-hospital mortality	567,858	1.3	437,467	1.0	130,391	9.5

*ED* emergency department, *IQR* interquartile range, *EMS* emergency medical service.

### Trends and epidemiologic characteristics of cancer-related ED visits

**Figure. 1:**
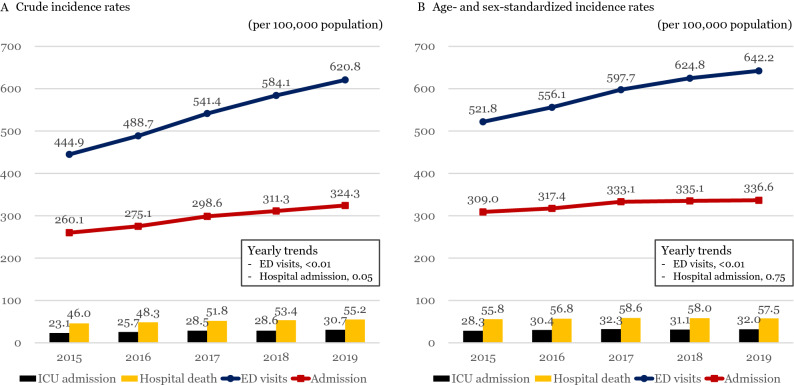
Trends of the crude and age- and sex-standardized incidence rates of cancer-related ED visits per 100,000 population from 2015 to 2019. **(A)** Crude incidence rate, **(B)** age-and sex-standardized incidence rate.

The crude incidence rate of cancer-related ED visits per 100,000 populations increased from 444.9 in 2015 to 620.8 in 2019 (p < 0.01). The age- and sex-standardized incidence rate of cancer-related ED visits per 100,000 populations also increased from 521.8 in 2015 to 642.2 in 2019 (p < 0.01). The annual age- and sex-standardized rates of hospital outcomes for cancer-related ED visits were presented in Fig. 1 (in 2015 and 2019, 309.0 and 336.6 for cancer-related hospital admission via ED, 28.3 and 32.0 for cancer-related ICU admission via ED, and 55.8 and 57.5 for in-hospital mortality). Analysis by cancer type was presented as supplement (Supplement Fig. 1).

For cancer-related ED visits, the most common cancer type was lung cancer, accounting for 14.7% of all cancer-related ED visits. Among them, 54.0% were hospitalized and 14.6% died in hospitals. The second most common ED visiting cancer type was liver cancer (13.1%), and followed by colorectal cancer (11.5%), stomach cancer (9.0%), and pancreas cancer (6.0%). Cancer types with high hospitalization rates were gallbladder and biliary tract cancer (59.4%), leukemia (54.9%), and non-Hodgkin lymphoma (54.1%) (Table 2).

**Table 2:** Cancer type and hospital outcomes among patients with cancer-related emergency department visits.

Cancer type (ICD-10 code)	Total		ED disposition (%)			In-hospital mortality (%)
	N	%	Ward	ICU	Death	
Total	1,372,119	100	49.7	5.1	0.8	9.5
Lung cancer (C33–C34)	201,367	14.7	54.0	5.6	1.3	14.6
Liver cancer (C22)	179,177	13.1	49.9	7.3	1.1	12.2
Colorectal cancer (C18–20)	157,654	11.5	48.2	5.4	0.6	7.4
Others	138,700	10.1	45.9	4.4	0.6	8.2
Stomach cancer (C16)	123,272	9.0	51.6	5.5	0.8	9.2
Pancreas cancer (C25)	82,077	6.0	51.2	3.3	0.9	10.8
Breast cancer (C50)	81,417	5.9	39.6	1.8	0.4	4.8
Gallbladder and biliary tract cancer (C23–24)	73,371	5.3	59.4	4.1	0.7	9.0
Leukemia (C91–95)	49,899	3.6	54.9	4.4	0.6	9.7
Non-Hodgkin lymphoma (C82–86, C96)	42,164	3.1	54.1	4.9	0.6	8.8
Prostate cancer (C61)	39,383	2.9	42.0	6.1	0.8	6.7
Ovarian cancer (C56)	31,159	2.3	48.2	2.1	0.5	5.2
Bladder cancer (C67)	30,907	2.3	46.7	5.0	0.5	6.4
Brain and CNS cancer (C70–72)	25,571	1.9	50.0	9.8	0.5	6.6
Cervix uteri cancer (C53)	23,002	1.7	45.4	3.2	0.6	5.5
Multiple myeloma (C90)	22,321	1.6	53.4	6.4	0.7	9.1
Esophagus cancer (C15)	20,697	1.5	50.6	5.1	1.1	10.5
Lip, oral cavity and pharynx cancer (C00–14)	18,182	1.3	41.0	4.6	1.2	8.7
Kidney cancer (C64)	17,495	1.3	50.0	5.7	0.6	8.1
Thyroid cancer (C73)	14,304	1.0	35.1	4.4	0.3	3.3

*ED* emergency department, *ICU* intensive care unit.

As for the primary reason for visiting the ED, 865,793 cases (63.1%) with a non-cancer diagnosis code were analyzed, excluding 506,326 cases (36.9%) that were diagnosed only with cancer code during cancer-related ED visits. The most common reason for ED visit was pneumonia, accounting for 3.6% of all cancer-related ED visits, of which 73.4% were hospitalized and 23.9% died in the hospital. The next most common primary reasons were gastroenteritis (2.7%), fever (2.6%), abdominal pain (2.4%), and ileus and intestinal obstruction (2.1%) (Table 3).

**Table 3:** Top 10 reason for emergency department visits and hospital outcomes among patients with cancer-related visits.

Diagnoses*	Total		ED disposition (%)			In-hospital mortality (%)
	N	%	Ward	ICU	Death	
Pneumonia	48,839	3.6	73.4	12.3	0.9	23.9
Gastroenteritis	37,714	2.7	50.5	2.4	0.1	4.6
Fever	36,329	2.6	53.2	2.8	0.1	4.7
Abdominal pain	33,051	2.4	49.4	2.3	0.1	5.6
Ileus and intestinal obstruction	28,622	2.1	78.7	3.7	0.1	5.1
Agranulocytosis	26,781	2.0	79.2	2.9	0.2	4.7
Diabetes	19,327	1.4	79.3	6.4	0.5	15.6
Dyspnea	16,171	1.2	55.9	8.6	1.4	21.1
Pleural effusion	14,410	1.1	63.7	4.2	0.4	14.5
Ascites	14,097	1.0	27.8	1.1	0.1	6.9

*ED* emergency department, *ICU* intensive care unit.

*Pneumonia, J12-18; fever, R50; abdominal pain, R10; ileus and intestinal obstruction, K56; gastroenteritis, A90, K52, K29; agranulocytosis, D70; dyspnea, R06; diabetes, E11-14; pleural effusion, K90-91, C78.2; ascites, R18, C78.6.

### Factors associated with ICU admission among cancer-related ED visits

Of the cancer-related ED visits, 69,948 cases (5.1%) were admitted to the ICU after ED treatment. Significantly increasing the odds for ICU admission were the over 65 years old compared to the 19–64 years old (AOR, 95% CI; 1.40, 1.38–1.42), the Medical Aid as health insurance compared to the NHI (AOR, 95% CI; 1.17, 1.14–1.20), transfer-in visit compared to the direct visit (AOR, 95% CI; 3.90, 3.83–3.98), EMS use (AOR, 95% CI; 3.88, 3.81–3.95), and night time (AOR, 95% CI; 1.16, 1.14–1.18). Compared with other diagnoses, pneumonia (AOR, 95% CI; 1.81, 1.77–1.87) and dyspnea (AOR, 95% CI; 1.35, 1.27–1.43) showed significant increases of odds for ICU admission (Table 4).

**Table 4:** Multivariable logistic regression model for intensive care unit admission of cancer-related emergency department visits.

Characteristics	Total	ICU admission		Odds ratio		
	N	N	%	Ratio	95% CI	
Total	1,372,119	69,948	5.1			
**Age**						
0–18	28,677	555	1.9	0.66	0.61	0.72
19–64	636,230	23,427	3.7	1.00		
65–120	707,212	45,966	6.5	1.40	1.38	1.42
**Sex**						
Male	805,819	46,151	5.7	1.00		
Female	566,300	23,797	4.2	0.81	0.80	0.83
**Insurance**						
National Health Insurance	1,233,364	60,958	4.9	1.00		
Medical Aid	125,803	7,939	6.3	1.17	1.14	1.20
Others	12,952	1,051	8.1	1.54	1.44	1.65
**Metropolitan**						
No	576,976	33,550	5.8	1.22	1.20	1.24
Yes	795,143	36,398	4.6	1.00		
**Route of ED visit**						
Direct	1,034,422	43,635	4.2	1.00		
Transfer-in	230,259	23,790	10.3	3.90	3.83	3.98
Others	107,438	2,523	2.3	0.95	0.91	0.99
**EMS use**						
No	1,123,809	43,580	3.9	1.00		
Yes	248,310	26,368	10.6	3.88	3.81	3.95
**Time of ED visits**						
Day time	930,296	45,417	4.9	1.00		
Night time	441,823	24,531	5.6	1.16	1.14	1.18
**Week of ED visits**						
Weekday	1,001,144	52,630	5.3	1.00		
Weekend	370,975	17,318	4.7	0.97	0.95	0.99
**Level of ED**						
Level 1	413,301	24,298	5.9	1.00		
Level 2	732,484	35,614	4.9	0.90	0.88	0.91
Level 3	226,334	10,036	4.4	0.83	0.81	0.85
**Primary diagnoses**						
Pneumonia	48,839	6000	12.3	1.82	1.77	1.87
Fever	36,329	1009	2.8	0.56	0.53	0.60
Abdominal pain	33,051	745	2.3	0.44	0.41	0.47
Ileus and intestinal obstruction	28,622	1062	3.7	0.64	0.60	0.68
Gastroenteritis	37,714	915	2.4	0.50	0.47	0.54
Agranulocytosis	26,781	775	2.9	0.73	0.68	0.79
Dyspnea	16,171	1388	8.6	1.35	1.27	1.43
Diabetes	19,327	1234	6.4	1.02	0.96	1.08
Pleural effusion	14,410	604	4.2	0.68	0.63	0.74
Ascites	14,097	149	1.1	0.25	0.22	0.30
Others	1,096,778	56,067	5.1	1.00		

*ED* emergency department, *IQR* interquartile range, *EMS* emergency medical service.

## Discussion

Using nationwide ED database, we investigated the trends and epidemiologic characteristics of cancer-related ED visits. In Korea, there were 1,372,119 cancer-related ED visits nationwide during the 5 years from 2015 to 2019, accounting for 3.1% of all ED visits. More than half of cancer-related visits were hospitalized, and 9.5% died in the hospital. Incidence rate of cancer-related ED visits increased during the study period (age- and sex-standardized incidence rate per 100,000 populations, 521.8 in 2015 and 642.2 in 2019, p < 0.01). The most common cancer types in cancer-related ED visits were lung cancer, liver cancer, and colorectal cancer. The most common primary reasons of ED visits were pneumonia, gastroenteritis, and fever. As the incidence and prevalence of cancer increase, cancer-related ED visits are also on the rise, and the increase in cancer-related ED visits with such high rates of hospitalization and mortality shows that the burden of the ED is increasing.

Cancer-related ED visits constituted 3.1% of all ED visits in Korea, slightly lower than that of 3.7 to 4.2% in the US studies^10,11^. More than 50% of cancer-related visits were hospitalized, similar to the US, which was about three times the hospitalization rate of non-cancer-related visits (54.8% versus 16.8%), and the ICU admission rates were also more than twice that of non-cancer-related visits (5.1% versus 2.3%). These results show that the burden on the ED for cancer-related visits is very high. The most common primary reason of cancer-related ED visits was pneumonia, and more than 70% of patients diagnosed with pneumonia were hospitalized. Diabetes, agranulocytosis, and ileus and intestinal obstruction were also hospitalized in more than 70%. A combination of cancer and chronic disease such as diabetes can increase the risk of hospitalization after an ED visit^20^. Agranulocytosis is a typical side effect that occurs after chemotherapy, and ileus is common after laparotomy^21,22^. The high hospitalization rate for these reasons means that cancer patients who visit the ED with these symptoms need close monitoring and treatment. Conversely, ascites had a relatively low hospitalization rate (27.8%) compared to others in the list of top 10 reasons. Some of the malignant ascites had to be managed through outpatient treatment rather than in the ED^23^.

The National Cancer Center in Korea publish national cancer statistics every year^18^. According to the most recently published 2017 cancer statistics, the 5-year cancer prevalence of Korea was 1.87 million, while top five cancers with the highest prevalence were thyroid, stomach, colorectal, breast, and prostate cancers^18^. Unlike these national prevalence data, the top five cancers that led to ED visits were lung, liver, colorectal, stomach, and pancreas cancer. Thyroid cancer accounted for 21.7% of the national cancer prevalence, but only 1.0% in cancer-related ED visits. In contrast, lung cancer accounted for the only 4.5% of the national cancer prevalence, but 14.7% in cancer-related ED visits. Cancer patients visit the ED when symptoms suddenly worsen or develop symptoms that cannot be treated on an outpatient basis. Because different cancer types have different symptoms and treatment side effects, there may be differences in the ED visits and hospital outcomes according to the cancer type. In terms of the management of medical resources, this is the reason why research on healthcare utilization for cancer patients by cancer type is needed in addition to nationwide prevalence statistics.

Traditionally, ED is a department that responds to sudden emergencies or time-sensitive conditions^13,24,25^. Cancer itself causes symptoms such as acute abdominal pain and dyspnea, and cancer treatments also cause side effects such as neutropenia^21,26^. For that reasons, cancer patients have to visit the hospital frequently, including ED^26,27^. Previously, little was known about the epidemiologic characteristics of cancer-related ED visits, as the main stage of the oncologist in charge of treating cancer patients are outpatient clinic and wards^27^. In response to the increasing trends of cancer-related ED visits, the American Academy of Emergency Medicine advocated in 2016 that four areas of research were needed to improve emergency care for cancer patients; (1) the collection of epidemiologic data, (2) care of patients with febrile neutropenia, (3) acute events such as dyspnea, and (4) palliative care in the ED setting^28^. This study, which presents epidemiologic data of cancer-related ED visits in Korea, is the first step. In addition, the studies of avoidable ED visits are also important, given the increasing number of cancer-related ED visits^29^. Some cancer patients may need hospice care rather than ED treatment^30^, and some cancer patients may not have visited the ED if they have been prescribed appropriate medications to control their symptoms^31^.

As cancer incidence and prevalence increase, number of cancer-related ED visits is also on an increasing trend. This is the first nationwide study on the epidemiologic trends of cancer-related ED visits in Korea, using a large, nationally representative database. Based on this epidemiologic data, further research is needed to reduce the burden of ED of cancer patients and improve cancer management in the ED.

This study has several limitations. Firstly, this study classified cancer-related ED visits based on the diagnosis codes entered into the NEDIS database. If there was a cancer diagnosis code in either one of the ED discharge codes or hospital discharge codes, it was classified as cancer-related ED visit. If the cancer diagnosis code is missing, the cancer-related ED visits may be misclassified. However, since patients with cancer diagnosis code have high coverage benefit in Korea, it is very unlikely that the cancer diagnosis was omitted. Secondly, NEDIS database lacks information on cancer staging or diagnosis/treatment status. It is unknown what cancer stage the patients are on, whether they are receiving active treatment, and when they were diagnosed with cancer. In-depth studies of various ED visits in cancer patients are needed to understand the associations between characteristics of cancer-related ED visits and their prognosis. Thirdly, the primary reasons for the ED visits were classified according to the order of non-cancer diagnosis code. If multiple non-cancer diagnoses were entered, only the first listed diagnosis code was used for the analysis, as the principal diagnosis coded first. However, there is a possibility of misclassification. In addition, since NEDIS database does not contain laboratory data, detailed information about the diagnosis is absent.

In conclusion, there were 1.37 million cancer-related ED visits over five years, which is increasing every year. The most common cancer types were lung cancer, liver cancer, and stomach cancer. The common primary reasons of cancer-related ED visits were pneumonia, gastroenteritis, and fever. More than half of cancer-related ED visits were hospitalized and in-hospital mortality rate was at 9.5%. Research and strategies are needed to reduce the growing burden of cancer-related ED visits and improve the ED care of cancer patients.

## Supplementary Information

Supplementary Figure S1.

## Data Availability

The data of this study were obtained from the National Emergency Medical Center under the Ministry of Health and Welfare in Korea but restrictions apply to the availability of these data and so are not publicly available.
