# Supplementary material for: Epidemiologic trends in cancer-related emergency department utilization in Korea from 2015 to 2019
Source: Sci Rep. 2021 Nov 9;11:21981. doi: 10.1038/s41598-021-01571-1 (PMC8578619; doi:10.1038/s41598-021-01571-1)
Supplement: Supplementary file 1 — Supplementary Figure S1. [file 41598_2021_1571_MOESM1_ESM.pptx]

## Slide 1
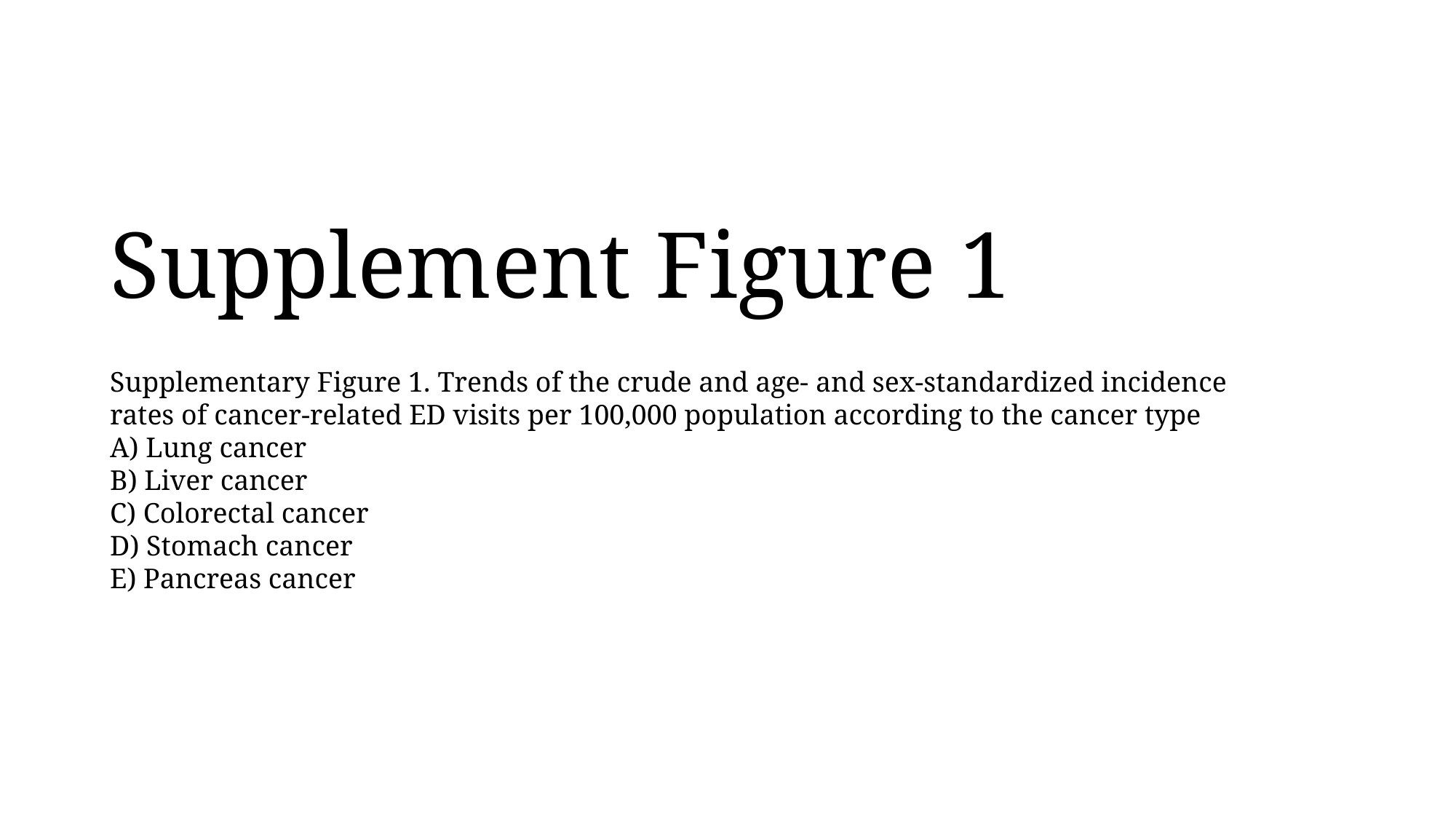

# Supplement Figure 1
Supplementary Figure 1. Trends of the crude and age- and sex-standardized incidence rates of cancer-related ED visits per 100,000 population according to the cancer type
A) Lung cancer
B) Liver cancer
C) Colorectal cancer
D) Stomach cancer
E) Pancreas cancer

## Slide 2
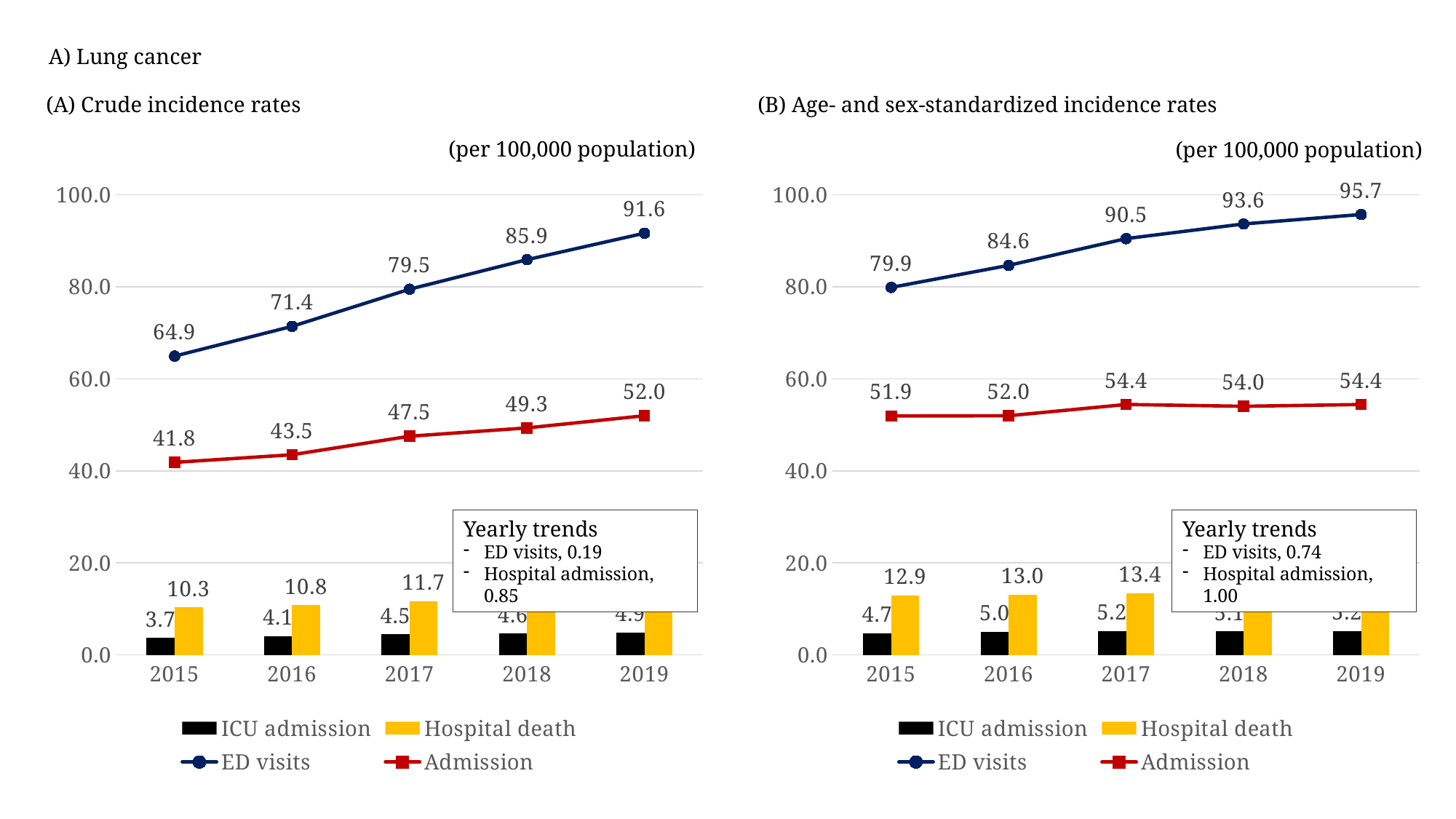

A) Lung cancer
(A) Crude incidence rates
(B) Age- and sex-standardized incidence rates
(per 100,000 population)
(per 100,000 population)
### Chart
| Category | ICU admission | Hospital death | ED visits | Admission |
|---|---|---|---|---|
| 2015 | 3.7054687006732787 | 10.319573319989459 | 64.9281332392338 | 41.841571625875865 |
| 2016 | 4.118328358193771 | 10.79178110394149 | 71.38696680939398 | 43.497764554737344 |
| 2017 | 4.477783479219806 | 11.653167990820505 | 79.45430537124768 | 47.51837882220006 |
| 2018 | 4.62955025946485 | 12.068019223725004 | 85.87572070767325 | 49.32469042757834 |
| 2019 | 4.91843927461611 | 12.505497086350662 | 91.62321907331403 | 51.973780881309715 |
### Chart
| Category | ICU admission | Hospital death | ED visits | Admission |
|---|---|---|---|---|
| 2015 | 4.6663 | 12.9304 | 79.8532 | 51.9109 |
| 2016 | 4.9858 | 13.0386 | 84.6376 | 51.9725 |
| 2017 | 5.1768 | 13.4292 | 90.4502 | 54.4136 |
| 2018 | 5.1017 | 13.2721 | 93.6396 | 54.0298 |
| 2019 | 5.1633 | 13.1157 | 95.6886 | 54.4112 |Yearly trends
ED visits, 0.19
Hospital admission, 0.85
Yearly trends
ED visits, 0.74
Hospital admission, 1.00

## Slide 3
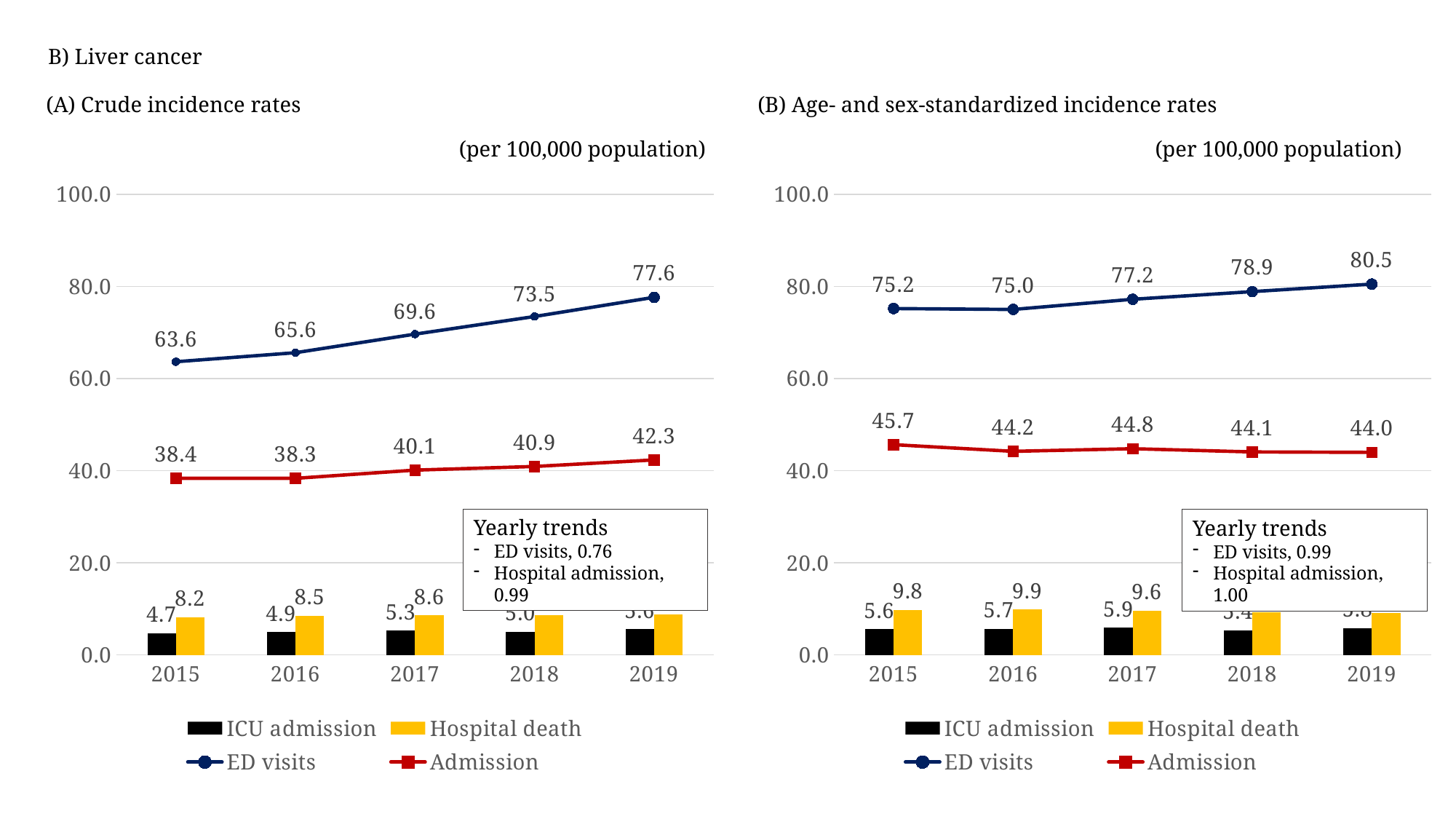

B) Liver cancer
(A) Crude incidence rates
(B) Age- and sex-standardized incidence rates
(per 100,000 population)
(per 100,000 population)
### Chart
| Category | ICU admission | Hospital death | ED visits | Admission |
|---|---|---|---|---|
| 2015 | 4.712304210972745 | 8.227396606579653 | 63.646527804096266 | 38.35395622275276 |
| 2016 | 4.947863381411898 | 8.549688800620798 | 65.6076119542375 | 38.33469161541508 |
| 2017 | 5.272228935210416 | 8.586647569785494 | 69.63207064263649 | 40.1204715047445 |
| 2018 | 5.009660701820911 | 8.56710458540969 | 73.46267815934813 | 40.917427156390175 |
| 2019 | 5.570984683327553 | 8.769431134384842 | 77.63147677249522 | 42.33169200631971 |
### Chart
| Category | ICU admission | Hospital death | ED visits | Admission |
|---|---|---|---|---|
| 2015 | 5.5819 | 9.7808 | 75.1876 | 45.6503 |
| 2016 | 5.7017 | 9.857 | 74.9929 | 44.2025 |
| 2017 | 5.8865 | 9.5973 | 77.2003 | 44.771 |
| 2018 | 5.3938 | 9.2419 | 78.8581 | 44.0694 |
| 2019 | 5.7838 | 9.113 | 80.4979 | 43.9789 |Yearly trends
ED visits, 0.76
Hospital admission, 0.99
Yearly trends
ED visits, 0.99
Hospital admission, 1.00

## Slide 4
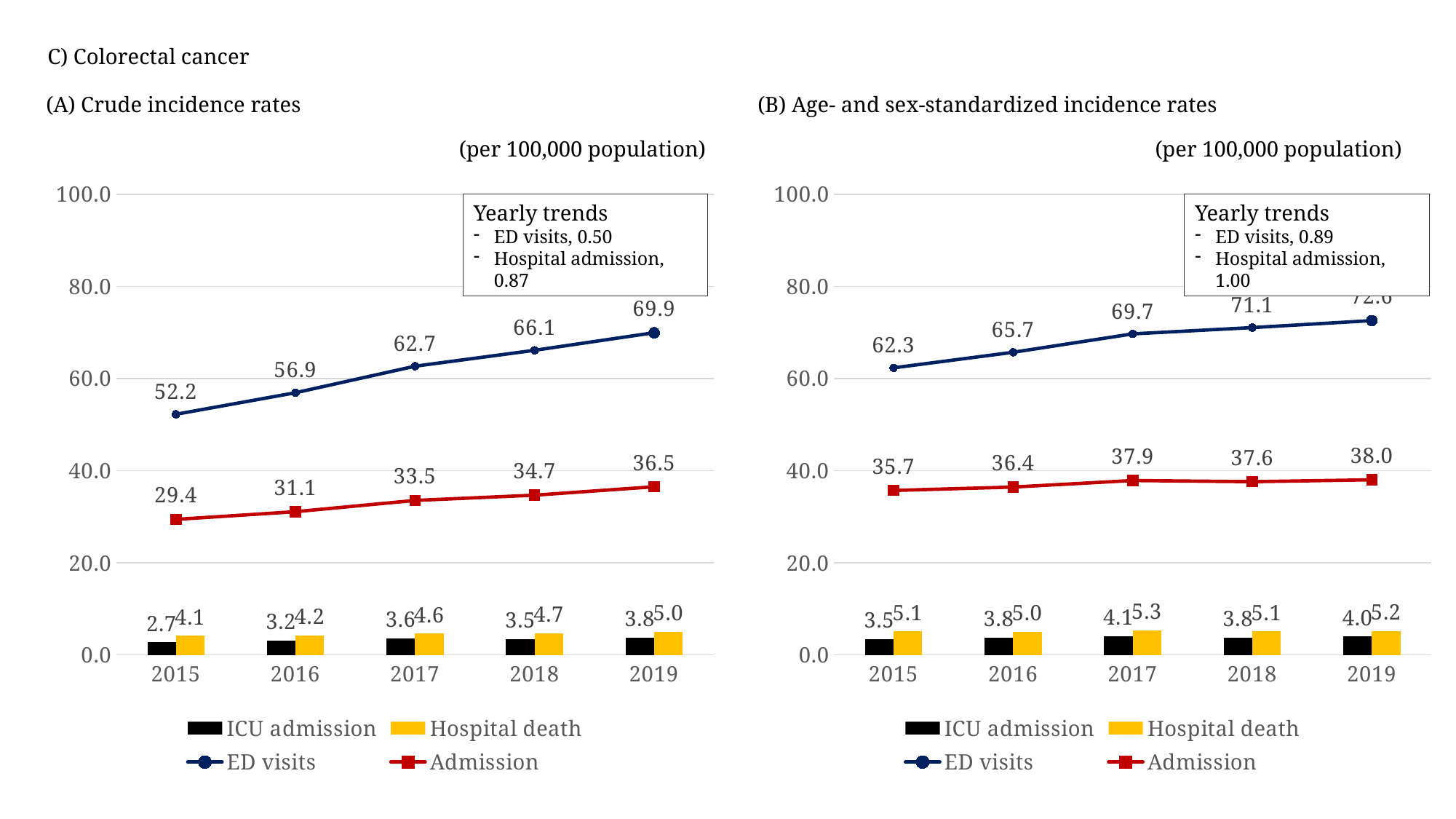

C) Colorectal cancer
(A) Crude incidence rates
(B) Age- and sex-standardized incidence rates
(per 100,000 population)
(per 100,000 population)
### Chart
| Category | ICU admission | Hospital death | ED visits | Admission |
|---|---|---|---|---|
| 2015 | 2.7359233944589776 | 4.147063222734448 | 52.24553856563701 | 29.420008380875235 |
| 2016 | 3.151841798123594 | 4.233758939254784 | 56.920971616764646 | 31.089955315941666 |
| 2017 | 3.572076620301763 | 4.643699606392292 | 62.67725698245333 | 33.532625278778134 |
| 2018 | 3.4677768048791444 | 4.668535945860343 | 66.12362269539648 | 34.67971733311122 |
| 2019 | 3.800346544465754 | 5.017782008479643 | 69.94507622689713 | 36.50748074647727 |
### Chart
| Category | ICU admission | Hospital death | ED visits | Admission |
|---|---|---|---|---|
| 2015 | 3.4658 | 5.0988 | 62.3232 | 35.7034 |
| 2016 | 3.8104 | 5.0301 | 65.694 | 36.4361 |
| 2017 | 4.1125 | 5.3082 | 69.6948 | 37.8523 |
| 2018 | 3.8146 | 5.0938 | 71.0556 | 37.6085 |
| 2019 | 3.986 | 5.2427 | 72.5786 | 38.0193 |Yearly trends
ED visits, 0.50
Hospital admission, 0.87
Yearly trends
ED visits, 0.89
Hospital admission, 1.00

## Slide 5
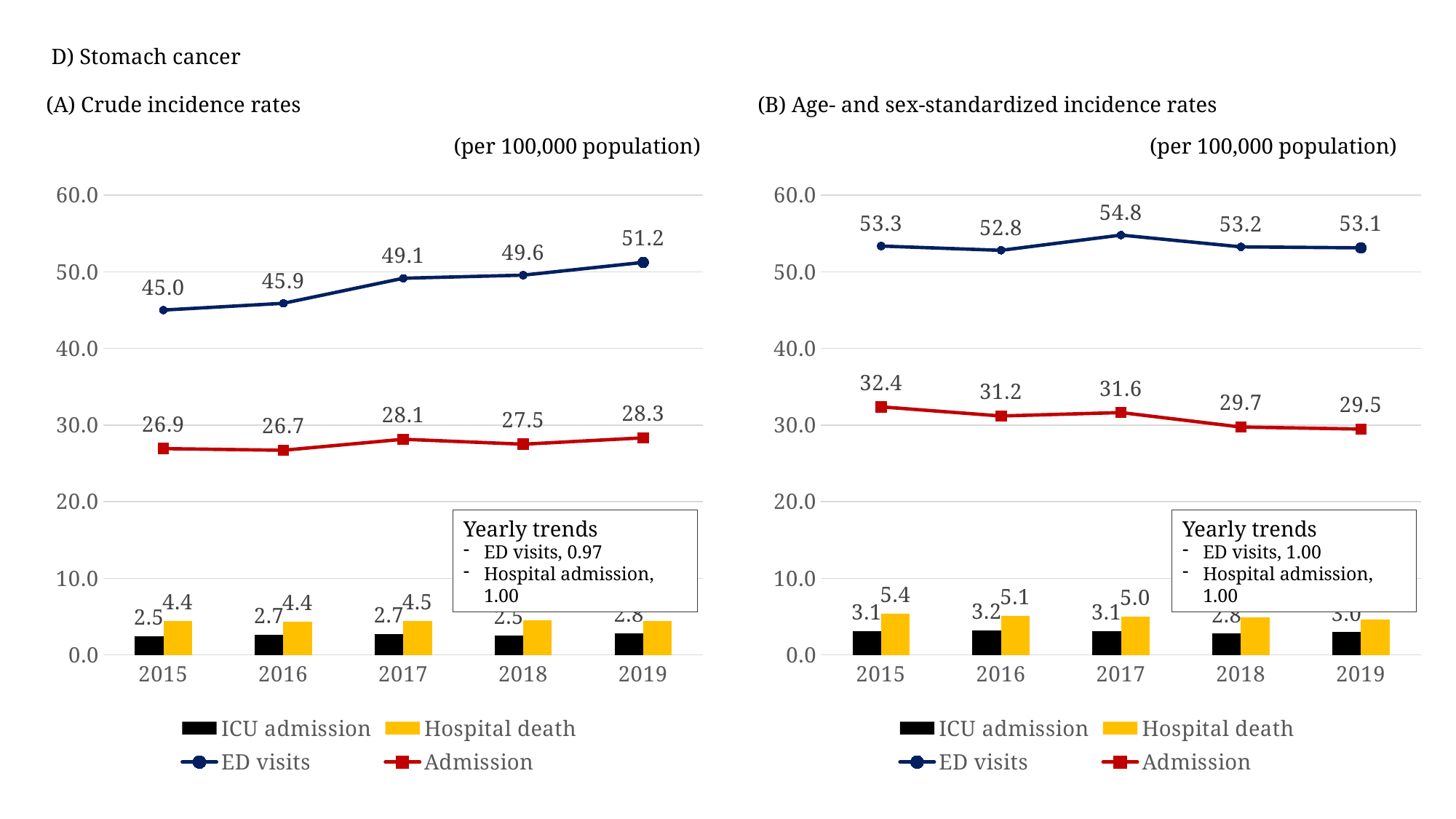

D) Stomach cancer
(A) Crude incidence rates
(B) Age- and sex-standardized incidence rates
(per 100,000 population)
(per 100,000 population)
### Chart
| Category | ICU admission | Hospital death | ED visits | Admission |
|---|---|---|---|---|
| 2015 | 2.47489196586282 | 4.449310140056315 | 44.99750047687302 | 26.925489991809698 |
| 2016 | 2.6685985180885052 | 4.360928223474544 | 45.87876484543654 | 26.709462587202548 |
| 2017 | 2.7366401211273614 | 4.4543600259719245 | 49.148260777365074 | 28.14718298620296 |
| 2018 | 2.526272478427977 | 4.506745347319046 | 49.552756692991984 | 27.500503183381095 |
| 2019 | 2.8458771406788657 | 4.474318817343843 | 51.21994484199231 | 28.33215811853121 |
### Chart
| Category | ICU admission | Hospital death | ED visits | Admission |
|---|---|---|---|---|
| 2015 | 3.0983 | 5.3873 | 53.3499 | 32.3764 |
| 2016 | 3.2095 | 5.1138 | 52.7925 | 31.1796 |
| 2017 | 3.1496 | 5.0232 | 54.781 | 31.6308 |
| 2018 | 2.7712 | 4.8933 | 53.2444 | 29.7389 |
| 2019 | 2.9802 | 4.6636 | 53.1147 | 29.4749 |Yearly trends
ED visits, 0.97
Hospital admission, 1.00
Yearly trends
ED visits, 1.00
Hospital admission, 1.00

## Slide 6
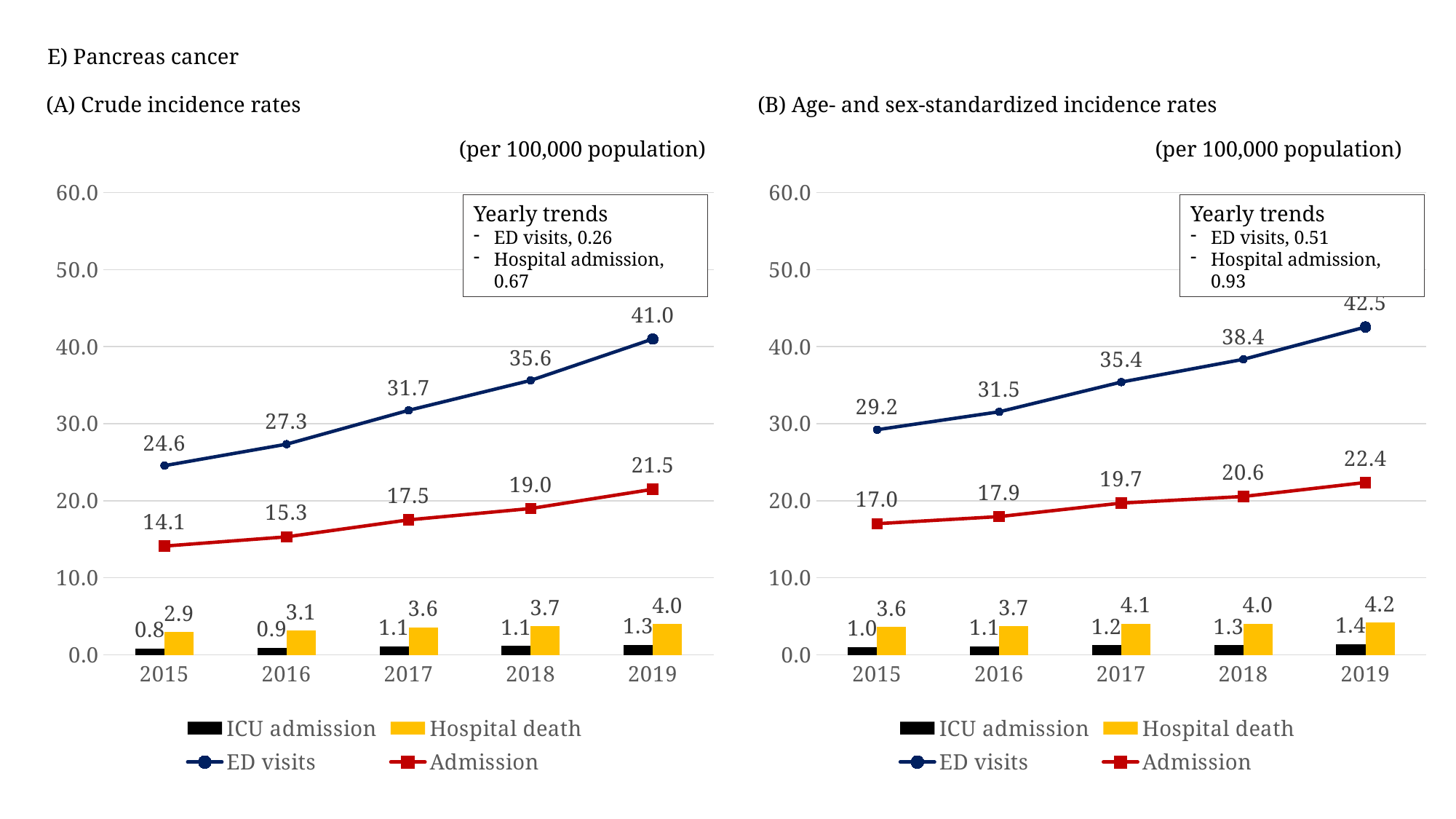

E) Pancreas cancer
(A) Crude incidence rates
(B) Age- and sex-standardized incidence rates
(per 100,000 population)
(per 100,000 population)
### Chart
| Category | ICU admission | Hospital death | ED visits | Admission |
|---|---|---|---|---|
| 2015 | 1.0207 | 3.603 | 29.2218 | 17.0254 |
| 2016 | 1.1008 | 3.7048 | 31.5485 | 17.9427 |
| 2017 | 1.2354 | 4.0578 | 35.4063 | 19.6971 |
| 2018 | 1.2547 | 4.0455 | 38.3564 | 20.556 |
| 2019 | 1.3772 | 4.1687 | 42.5476 | 22.3695 |
### Chart
| Category | ICU admission | Hospital death | ED visits | Admission |
|---|---|---|---|---|
| 2015 | 0.8262724168344546 | 2.9400381957672517 | 24.562468638202375 | 14.119248852035787 |
| 2016 | 0.9214881979616465 | 3.1401030949648465 | 27.347265458827803 | 15.317051171638495 |
| 2017 | 1.0872386215891157 | 3.5915961646749968 | 31.742683059752604 | 17.52269498385187 |
| 2018 | 1.1481284643472829 | 3.709488060531204 | 35.625120228201936 | 18.991877127564646 |
| 2019 | 1.3167781978774218 | 3.995136218707976 | 40.995434841018074 | 21.489196862401947 |Yearly trends
ED visits, 0.26
Hospital admission, 0.67
Yearly trends
ED visits, 0.51
Hospital admission, 0.93
